# Epitope mapping of diverse influenza Hemagglutinin drug candidates using HDX-MS

**DOI:** 10.1038/s41598-019-41179-0

**Published:** 2019-03-18

**Authors:** Cristina Puchades, Başak Kűkrer, Otto Diefenbach, Eveline Sneekes-Vriese, Jarek Juraszek, Wouter Koudstaal, Adrian Apetri

**Affiliations:** Janssen Vaccines and Prevention, Janssen Pharmaceutical Companies of Johnson & Johnson, Archimedesweg 6, 2333 CN Leiden, The Netherlands

## Abstract

Epitope characterization is critical for elucidating the mechanism of action of drug candidates. However, traditional high-resolution epitope mapping techniques are not well suited for screening numerous drug candidates recognizing a similar target. Here, we use Hydrogen-Deuterium Exchange Mass Spectrometry (HDX-MS) to explore the conformational impact of diverse drug molecules binding on Hemagglutinin (HA), the major surface antigen of influenza viruses. We optimized a semi-automated HDX-MS workflow to systematically probe distantly related HA subtypes in complex with 4 different drug candidates, ranging from a monoclonal antibody to a small synthetic peptide. This fast, cost-effective HDX-MS epitope mapping approach accurately determined the main antigenic site in all cases. Moreover, our studies reveal distinct changes in the local conformational dynamics of HA associated to the molecular mechanism of neutralization, establishing a marker for broad anti-HA activity. Taken together, these findings highlight the potential for HDX-MS epitope mapping-based screening to identify promising candidates against HA at early stages of drug discovery.

## Introduction

An important step in understanding the mechanism of action for protein drug candidates is to elucidate their interaction with the target antigen. The most powerful techniques for mapping epitopes providing the highest level of structural detail of the interactions are undoubtedly NMR spectroscopy^1,2^, X-ray crystallography^3,4^ and more recently Cryo-Electron Microscopy (cryo-EM)^5,6^. However, these high-resolution methods are quite expensive, laborious and therefore low throughput. In addition, NMR is not suitable for protein complexes exceeding a certain size, while X-Ray crystallography and Cryo-EM are often not successful either because of the intrinsic nature of the proteins or difficulties in reaching the desired level of monodispersity^7,8^.

Hydrogen-deuterium exchange mass spectrometry (HDX-MS) has emerged as an alternative method for epitope/paratope mapping with important applications in drug discovery^7–11^. HDX-MS measures the kinetics of hydrogen-deuterium exchange at protein backbone amides. The rate of exchange for a backbone amide hydrogen is directly affected by both involvement in H-bonding and solvent exposure^12^. When a certain exposed region on an antigen protein engages in an interaction with another protein, the level of solvent accessibility decreases significantly, consequently reducing the rate of amide H exchange and the deuterium incorporation of the affected residues. Thus, decreased deuteration is indicative of protein-protein interactions and when coupled with proteolytic digestion can provide details regarding the regions of the proteins involved in the interaction. In addition, HDX-MS can reveal the allosteric effects of protein binding in solution, as it reports on the local conformational dynamics of protein complexes in a near-native environment. Unlike X-Ray Crystallography, HDX-MS requires relatively low amounts and concentrations of proteins, and is suitable for proteins with higher levels of polydispersity^13–16^. Fast, cost-effective HDX-MS studies are enabled by recently developed instrumentation that integrates complete on-line semi-automated HDX-MS sample management and data collection, and software for automated data analysis^17,18^. However, the application of HDX-MS for epitope mapping of large, highly glycosylated antibody-antigen complexes can be challenging.

**Figure 1:**
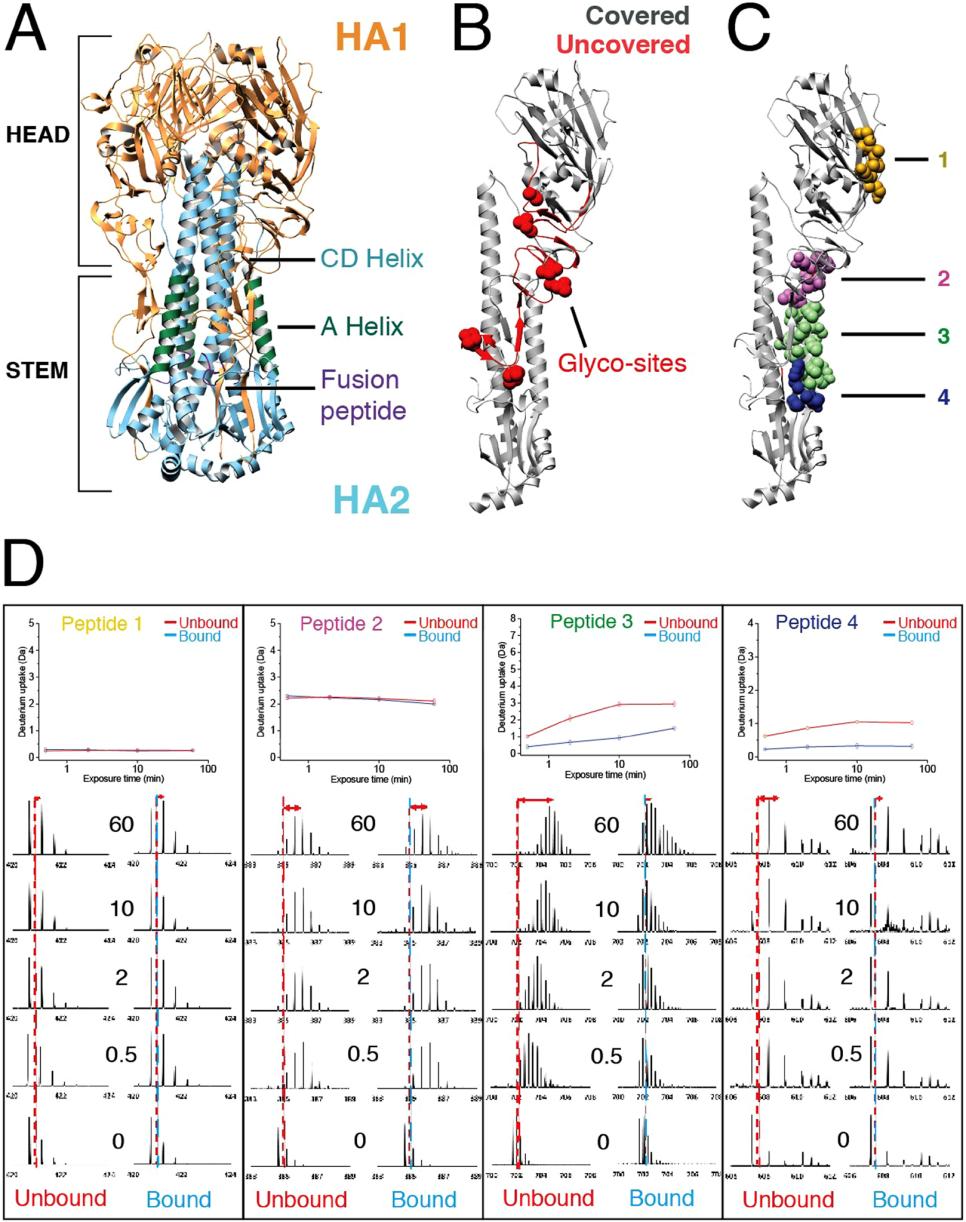
HDX-MS reveals the conformational dynamics of free- and Fab-bound HA trimers in solution. (**A**) Atomic model of the HA trimer (PDB: 3UBQ) showing the HA head and stem, which mainly correspond to HA1 (orange) and HA2 (blue), respectively. In HA2, we highlight the CD Helix (residues 75–126) that forms a trimeric coiled coil driving oligomerization, and the highly conserved components of the fusion machinery required for HA function: The A Helix (residues 38–58, shown in green) and the fusion peptide (residues 1–10, shown in purple). (**B**) Atomic model of a single protomer of HA (PDB: 3UBQ) with the areas covered by our list of highly reproducible peptides shown in grey, whereas uncovered regions are colored red. All uncovered regions are found around the 7 glycosylation sites of HA1, which are highlighted as red spheres. (**C**) The residues of four representative peptides from our peptide library are shown as spheres in the atomic model of the HA monomer: Peptide 1 (^119^ERFEIF^124^, yellow) corresponds to an exposed β strand in HA1, Peptide 2 (^58^KMNTQF^63^, pink) is a flexible loop in the HA2 subunit, Peptide 3 (^39^KSTQNAIDEITNKVNSVIE^57^, green) covers the entire A Helix, and Peptide 4 (^17^MVDGW^21^, blue) is directly adjacent to the N-terminal fusion peptide (**D**) The deuterium uptake graphs for Peptides 1–4 show the relative deuterium incorporation of each peptide as a function of exposure time (calculated as the difference between the mass centroid at t = 0 and the different labeling time points). Below, we show the mass spectra of each peptide for a selected charge state for each labeling time point in the bound and unbound states. Peptides originating from free HA show distinct profiles of deuterium incorporation that closely match the expected behavior based on the atomic model of HA. While binding of Fab CR6261 does not alter deuterium uptake profiles of Peptides 1 and 2, deuterium incorporation of Peptides 3 and 4 is significantly lower at all labeling time points in bound HA.

Influenza remains a major threat for public health, as this highly variable virus causes thousands of deaths each year and current flu vaccines can protect against seasonal strains, but are not effective against pandemic outbreaks^19^. In the last decade, various broadly neutralizing human monoclonal antibodies (bnAbs) against influenza viruses have been described that target the major envelope protein of influenza A viruses, Hemagglutinin (HA)^20–27^. From a structural perspective, mature HA assembles as a 200 kDa homotrimer. Every protomer comprises two disulphide-linked chains known as HA1 and HA2 that correspond to the highly variable, heavily glycosylated HA head and the conserved HA stem, respectively (Fig. 1A). The broadly neutralizing antibodies target the highly conserved fusion machinery in the HA stem, and inhibit pH-induced conformational changes that are required for viral fusion in the endosomal compartments of host cells^20–23,26,28^. Given their ability to neutralize wide spectra of viruses within and across influenza virus subtypes, several bnAbs are being evaluated in clinical trials as passive immunotherapy and the epitopes recognized by these drug candidates are being used for vaccine design^29^. In addition, efforts have been undertaken to target the conserved stem region of HA with new antiviral molecules such as composites of llama single-domain antibodies^30^, small proteins^31–33^ and peptides^34,35^.

In this study, we use HDX-MS to determine the epitope of bnAb CR6261^22,27^, llama single-domain antibodies SD38 and SD84^36^ and cyclic peptide P3^34^. Our HDX-MS studies correctly determine the epitope of all drug candidates tested and reveal precisely how the different therapeutic molecules distinctly alter the conformational dynamics of trimeric HA in solution. Moreover, we identify distinct changes in HA conformational dynamics linked to broad anti-HA activity. Our HDX-MS studies provide new insights into the molecular mechanisms underlying HA neutralization and establish the potential of HDX-MS for drug discovery pipelines aimed at identifying broadly neutralizing drug molecules against HA.

## Materials and Methods

### Reagents

TCEP (tris-(2-carboxyethyl) phosphine), formic acid (MS grade ~98%), sodium phosphate monobasic, sodium phosphate di-basic and sodium chloride were purchased from Sigma-Aldrich (St. Louis, MO, USA). Deuterium oxide (99.9%) was purchased from Cambridge Isotope Laboratories (Andover, MA, USA). Guanidine hydrochloride (8.0 M) was purchased from Thermo Scientific (Waltham, MA, USA). Acetonitrile and water were UPLC/MS grade and purchased from BioSolve B.V. (Valkenswaard, The Netherlands). All chemicals were used without further purification unless otherwise specified.

### Antigens and drug candidates

Hemagglutinin proteins of Influenza A/California/07/2009 (H1N1) and B/Brisbane/60/2008 (Victoria lineage) strains were expressed and purified as previously described^21,22^, and buffer exchanged into PBS, pH 7.4 to a final concentration of 5.9 mg/ml and 7.3 mg/ml, respectively. Fab-CR6261 was expressed and purified as previously described^22^, formulated in 10 mM sodium citrate, 150 mM NaCl, 0.01% Tween80 at 0.7 mg/ml, and buffer exchanged to PBS, pH 7.4 to a final concentration of 10.9 mg/ml. SD38 and SD84 were expressed, purified and formulated in PBS buffer, pH 7.4 at 5.7 mg/ml and 8.5 mg/ml, respectively^30^. Cyclic peptide P3 was provided as dry powder^34^ and dissolved in DMSO to a final concentration of 25 mM and further diluted in PBS buffer, pH 7.4.

### Binding studies by SEC-MALS

For size exclusion chromatography (SEC) based binding studies, 18.8 µM HA was injected either alone or mixed with Fab-CR6261 (1:1.1 HA: Fab monomer unit ratio) on an analytical column (TSK-Gel 3000SWxl; Tosoh Bioscience) equilibrated with 150 mM sodium phosphate and 50 mM NaCl, pH 7.0. Samples were separated at a flow rate of 1.0 ml/min. For molar mass determination, in-line UV (Agilent 1260 Infinity MWD; Agilent Technologies), refractive index (Optilab T-rEX; Wyatt Technology), and eight-angle static light-scattering (Dawn HELEOS; Wyatt Technology) detectors were used. Astra Software, including the protein conjugate analysis function, was used for data analysis.

### HDX-MS sample preparation

The HA protein concentration was adjusted to 60 μM for HA A/California/07/2009 and 90 μM for HA B/Brisbane/60/2008 in 100 mM sodium phosphate buffer, pH 7.4. In both cases, 3 µl of unbound HA samples were diluted 20-fold in either aqueous PBS, pH 7.4 (for non-deuterated experiments) or PBS in D_2_O, pD 7.4 (for deuterated experiments) and incubated at 25 °C. At specific time points, samples were withdrawn from the continuous labelling reaction and quenched by 1:1 volume addition of ice cold quenching buffer containing 100 mM potassium phosphate, 4 M guanidine hydrochloride and 0.5 M TCEP, pH 2.5 after 0.5, 2, 10 and 60 min for the HA A/California/07/2009 strain, and 0.3, 0.7, 2, 13 and 60 and 240 min for HA B/Brisbane/60/2008. Each experimental condition was tested in triplicate. The drug-bound complexes of HA A/California/07/2009 was prepared with 1.2-fold molar excess of Fab-CR6261, SD38 and P3, respectively. Similarly, a 1.4-fold molar excess of SD84 was incubated with HA B/Brisbane/60/2008. In all cases, 3 μl of protein/complex solution were diluted 20-fold in either aqueous PBS, pH 7.4 or deuterated PBS pD 7.4. Labelling and quenching conditions were identical to unbound HA samples. After a brief vortex, the quenched samples were snap frozen in liquid N_2_ and stored at −80 °C until MS analysis. Once the method was optimized, a LEAP PAL autosampler (LEAP Technologies, Carrboro, NC, USA) was used for semi-automated sample preparation and injection.

### HDX-MS workflow and data collection

Samples collected at different time points were thawed and injected into a Waters nano-ACQUITY UPLC system equipped with HDX technology (Waters Corporation, Milford, MA). Online digestion was performed on an immobilized pepsin column (Poroszyme Immobilized Pepsin Cartridge) at 25 °C with 0.05% formic acid in H_2_O, pH 2.5 at a flow rate of 125 μl/min. Peptides were trapped and desalted online on an ACQUITY UPLC BEH C18 1.7 µm VanGuard Pre-column 3/Pk 2.1 × 5 mm (Waters Corporation, Milford, MA, USA) for 4 min at approximately 0 °C. Peptide separation was performed using a 12-min linear acetonitrile-water gradient (8–95% containing 0.1% formic acid) at a flow rate of 40 μl/min on an ACQUITY UPLC BEH C18 1.7 µm 1.0 × 100 mm analytical column (Waters Corporation, Milford, MA, USA) at 0 °C. The eluent was directed to a Waters Synapt Q-TOF G2 ESI mass spectrometer lock-mass corrected using 2 ng/µl Leu-Enkephalin solution. The following instrument configuration was used: capillary voltage +1.5 kV, sampling cone voltage 30 V, trap collision voltage 4 V (low energy) and 20–40 V ramping (elevated energy). The source and desolvation temperatures were set to 100 °C and 250 °C, respectively. Mass spectra were acquired in resolution mode over an *m/z* range of 50–2000. Peptides were identified using MS^E^ acquisition. To eliminate peptide carryover, two blank injections of 0.1% formic acid in H_2_O were injected after each sample run.

### HDX-MS data analysis and interpretation

The identification of peptides was performed for the unlabelled HA samples using ProteinLynx Global Server (PLGS) 2.5.2 software (Waters Corporation, Milford, MA, USA). Deuterium uptake for each peptide was calculated and compared to the results for the non-deuterated, unlabelled sample using DynamX 3.0 software (Waters Corporation, Milford, MA, USA). Only peptides observed in both the non-deuterated and deuterated samples above the pre-set thresholds of 2000 intensity, 0.2 minimum products per amino acid and present in 2 out of 3 of the replicates were further considered. Absolute deuterium incorporation per peptide at a given time point corresponding to the centroid value across the backbone amides was determined by comparison with the non-deuterated sample at t = 0. Results were averaged across triplicate measurements. For each peptide, the 3-fold value of the Standard Deviation calculated from triplicate repeats was used as a threshold (~0.3 Da for most peptides) above which deuterium uptake differences between the drug-free and -bound samples were considered significant. X-Ray crystal structures of the corresponding HA strains were used to visualize the outcome of the HDX experiments by projecting the heatmap results (with positive b-values) from each labelling time point of bound and unbound states of HA on the corresponding PDB structure as indicated in the text and Figures.

## Results and Discussion

### HDX-MS reveals the conformational dynamics of HA trimers in solution

HDX-MS analysis is reliant on the identification of highly reproducible peptides covering the entire sequence of the protein of interest. This presents a challenge for large, highly-glycosylated antigens like HA, because digestion of larger complexes produces an increasing number of peptides and overlapping mass-spectra complicate proper HDX-MS identification. In addition, the presence of glycans hampers enzymatic cleavage, increases heterogeneity of the generated peptides, and causes difficulties for the MS detection of glyco-peptides. Therefore, we optimized a commercially available, semi-automated HDX-MS workflow (Waters Corporation, Milford, MA, USA) for unbound, non-deuterated HA trimer to obtain a peptide list covering the complete HA sequence (Fig. S1). Briefly, we found that slightly increasing the amount of HA injected (36 μg HA) in combination with high concentrations of chaotropic and reducing agents in our quench solution (4 M Guanidine and 0.5 M TCEP) maximized the number of HA peptides produced. Increasing the digestion flow rate (125 μl/min) minimized damage to the immobilized pepsin enzyme without decreasing the number of generated peptides, and an empirically optimized reversed phase chromatography gradient significantly improved peptide separation and peptide identification by MS. Following the identification of peptides, unbound HA was subjected to deuterium labeling by incubating it with 20-fold excess of deuterated buffer at 25 °C and pH 7.4. The labeling was quenched at time intervals of 0.5, 2, 10 and 60 minutes (Fig. S1). Digestion after deuteration preserved a set of 119 highly reproducible staggered peptides, which constituted our reference peptide library (Fig. S2). No glyco-peptides were present in our final peptide library, resulting in a lack of coverage of the residues immediately around the 7 glycosylation sites of HA (Figs 1B and S2A). Analysis of HA samples that were deglycosylated by PNGase treatment confirmed that the loss of coverage in these areas is indeed due to the presence of glycans (Fig. S3). However, PNGase treatment resulted in decreased stability of the antigen leading to aggregation (data not shown). We thus performed all subsequent experiments with fully glycosylated HA, since most of the HA sequence (~80%) remained robustly covered, and our library contained an abundance of partially overlapping peptides covering the HA stem (Fig. S2A).

Importantly, groups of staggered peptides originating from the same regions presented similar deuterium uptake profiles, thereby strengthening our HDX-MS data (Fig. S2). Based on the measured level of deuterium uptake in several unstructured peptides, we calculated an average 45–50% back-exchange for our HDX-MS system under the reported experimental conditions (Fig. S2B). While a fully deuterated HA control would be needed to draw structural inferences directly from our HDX-MS data^37,38^, our deuterium uptake results for each region are in close agreement with a recent back exchange-normalized HDX-MS study of HA spikes on a viral envelope^39^. Moreover, deuterium uptake of individual peptides closely matched the expected trends based on their location within the 3D structure of the HA trimer previously determined by X-Ray Crystallography. For instance, representative Peptide 2 covers residues ^58^KMNTQF^63^, which correspond to a flexible exposed loop in the atomic structure of HA2 (Fig. 1C). As expected, this peptide was highly deuterated at early time points and no further deuteration was found for later time points (Fig. 1D). In contrast, peptides stemming from the highly ordered CD Helix, which forms a trimeric coiled coil at the inter-subunit interface of the HA trimer (Fig. 1), presented minimal deuterium incorporation at all labeling time points. This deuterium uptake profile reflects the conformational rigidity and low solvent exposure of this conserved secondary structure element in the HA stem (Fig. S2). While X-Ray crystallography provides a static atomic model, HDX-MS reports on the dynamic behavior of proteins in solution, as progressive incorporation of deuterium over time reflects local conformational dynamics. For example, Peptide 1 covering HA1 residues ^119^ERFEIF^124^ originates from a β-strand in the HA head, whereas Peptide 3 covering HA2 residues ^39^KSTQNAIDEITNKVNSVIE^57^ corresponds to a highly conserved component of the fusion machinery in the HA stem, known as the A Helix (Fig. 1). Both of these secondary structure elements are exposed on the HA surface and show protection at lower time points (Fig. 1C and D). However, deuteration levels for Peptide 1 remain low throughout the course of the experiment, which results in a very low, flat deuterium uptake graph, indicative of a highly ordered structure with minimal conformational dynamics in solution. In contrast, Peptide 3 incorporates very high levels of deuterium over time, giving rise to a steep deuterium uptake graph, which indicates high conformational flexibility of the A Helix in solution. In fact, at longer labeling time points (t = 60 min) this region incorporates the highest level of deuterium in the entire molecule, revealing unexpectedly high “conformational breathing” of this secondary structure element (Figs 1 and S2B). Importantly, the A Helix is known to undergo major pH-dependent conformational rearrangements that are required for fusion, which suggests that the conformational flexibility of the A Helix reported by HDX-MS is likely important to enable these essential structural changes. Thus, our HDX-MS analysis provides unique insights into the local conformational dynamics of HA trimers in solution with important implications for HA function.

### Binding of Fab CR6261 induces changes in the local conformational dynamics of HA that pinpoint the epitope

CR6261 is a potent broadly neutralizing monoclonal IgG1 antibody targeting the HA stem and the precise HA-Fab interactions have been previously characterized in atomic detail by X-ray crystallography^22^. Thus, we chose to characterize the effects of Fab CR6261 binding to HA to investigate the feasibility of HDX-MS as an epitope mapping alternative for molecules targeting HA. To ensure quantitative binding, the HA trimer was incubated with Fab CR6261 in a 1:1.1 molar monomer ratio, and Size Exclusion Chromatography coupled with Multi Angle Light Scattering (SEC-MALS) confirmed that 3 Fabs are bound per HA trimer under our HDX-MS experimental conditions (Fig. S4). Comparison of deuterium incorporation for a given HA peptide originating from free and Fab-bound HA complexes reveals the local effects caused by binding of the drug molecule (Fig. 1C and D). For example, deuteration levels of Peptides 1 and 2 are essentially identical for all labeling time points in free and bound HA, indicating that the local structure of the corresponding region of HA is not affected. In contrast, a clear reduction in deuterium incorporation in the HA-Fab complex was observed for Peptides 3 and 4, which suggests that these regions are involved in Fab binding.

**Figure 2:**
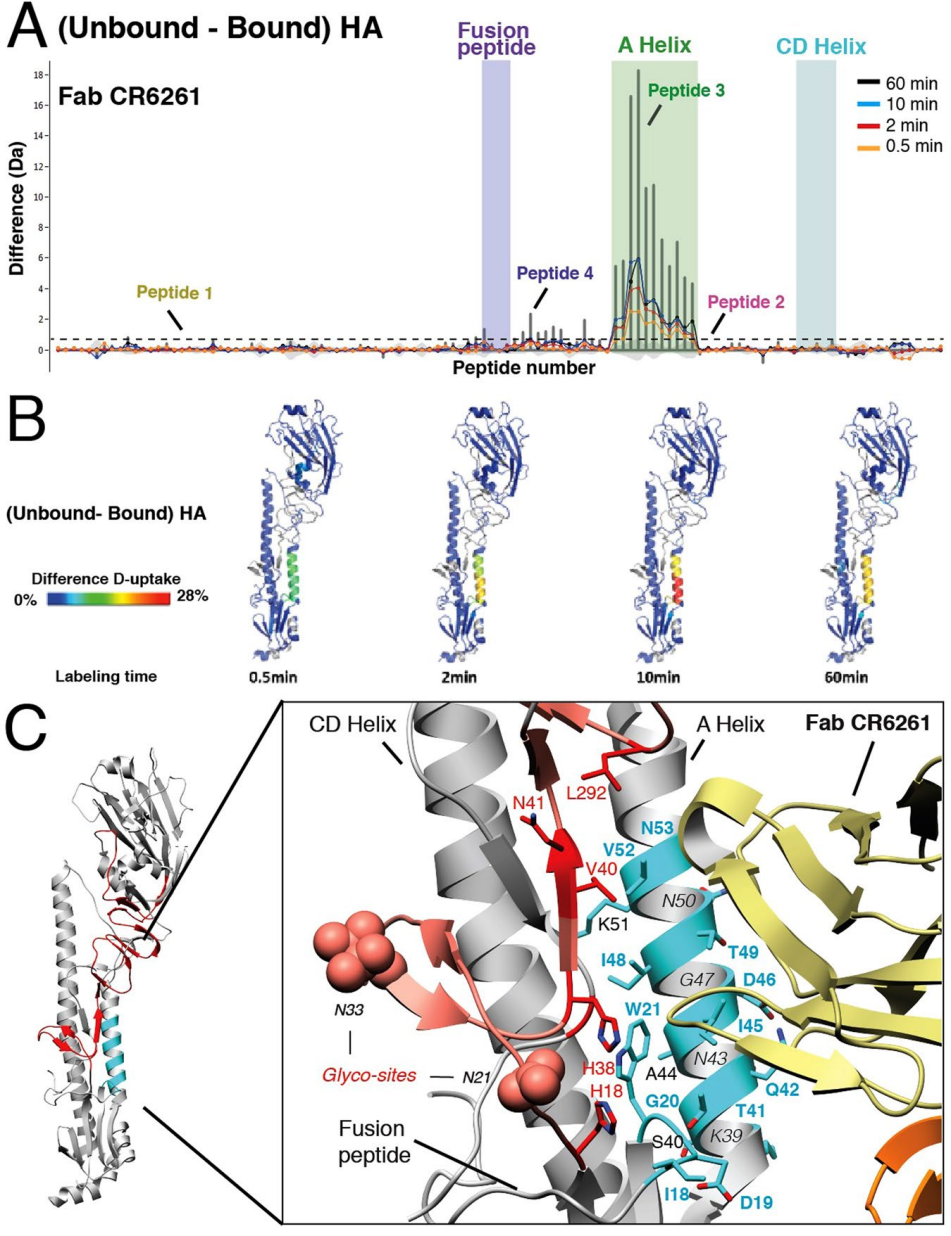
Binding of Fab CR6261 induces discrete local changes in deuterium incorporation that pinpoint the epitope. (**A**) Bar plot representing differences in deuterium uptake between free- and Fab CR6261-bound HA, with each bar corresponding to a unique HA peptide from the peptide library (ordered from N- to C- terminus based on the first amino acid of each peptide). The Y-axis represents the total difference in D uptake for a given peptide, calculated as the sum of the mass differences between free and bound HA at each time point. Positive bars indicate protection of the corresponding peptide upon binding and reveal that two distinct regions are affected by Fab CR6261. Deuterium uptake differences are shown for each peptide and labeling time point: 0.5 min (orange), 2 min (red), 10 min (blue) and 60 min (black). Light grey shades show the differential uptake errors for each peptide, and a dashed black horizontal line represents the threshold above which deuterium uptake differences between free and bound HA were considered significant. This threshold corresponds to three times the average standard deviation between triplicate repeats, and is approximately 0.3 Da for all measured peptides. (**B**) Deuterium uptake differences for each labeling time point projected on the monomer of HA A/California/07/2009 (PDB: 3UBQ) in a rainbow color scheme, where blue corresponds to HA regions that are not affected by Fab CR6261 binding, while red corresponds to the highest observed differences. Regions encompassing residues 18–21 and 39–54 in HA2 show protection upon Fab binding, and are therefore identified as the epitope by HDX-MS. (**C)** Atomic model of HA with the epitope determined by HDX-MS highlighted in cyan, and the uncovered regions shown in red. The close-up view shows the atomic details of the epitope determined in the co-crystal structure of HA SC1918/H1 in complex with Fab CR6261 (PDB: 3GBN), and HA residues involved in direct contacts are shown as sticks. The residues identified by HDX-MS are colored cyan, while those not detected are highlighted in red. Residues identified by both HDX-MS and X-Ray crystallography are labeled in green, and residues buried within the HA structure are labeled in italic. The 19 residues identified by HDX-MS as the epitope contain 16 residues seen to contact Fab CR6261 in the X-Ray structure, and the only 5 residues missed by HDX-MS localize to uncovered regions around glycosylation sites of HA1, which are shown as red spheres.

Automated software analysis^18^ allowed us to more broadly identify the effects of Fab CR6261 binding to the HA trimer by determining the differential deuterium uptake between the free and bound state for the entire peptide library (Fig. 2A). Notably, all peptides exhibiting differences in deuterium uptake correspond to two discrete regions. To rapidly visualize the effects of Fab binding on the local conformational dynamics of the antigen, we systematically mapped the deuterium uptake differences calculated for each time point onto the atomic structure of HA (Fig. 2B). The two areas affected correspond to the A Helix and the region directly adjacent to the fusion peptide, both of which are important components of the highly conserved fusion machinery. Further analysis of the overlapping peptides allowed us to conclude that only amino acid regions 18–21 and 39–54 of HA2 are affected by complex formation. These data suggest that these regions are actively engaged with Fab CR6261, and are therefore the epitope identified by HDX-MS. Comparison of our HDX-MS results with a high-resolution X-ray co-crystal of Fab CR6261 in complex with HA (Fab-CR6261-HA A/Brevig Mission/1/1919, PDB: 3GBN) confirmed that the two-amino acid sequence stretches identified as protected by HDX-MS indeed correspond to the epitope (Fig. 2C). In fact, from our final list of 24 amino acids, 16 are directly involved in interactions with Fab CR6261 in the co-crystal structure. However, the epitope in the X-ray structure also includes HA1 residues H^18^, H^38^, V^40^, N^41^, S^291^, L^292^ and T^319^, which were not detected by HDX-MS, because the presence of glycan sites in these specific regions hampered coverage of these areas (Fig. 2C). Despite the limitations posed by this large, highly glycosylated antigenic complex, we show that HDX-MS precisely determines the main recognition site of Fab CR6261.

Importantly, our HDX-MS data show that complex formation decreases deuterium incorporation in the A Helix by approximately 20% after 60 min labeling, indicating that Fab binding abolishes the significant conformational breathing observed for the A Helix in unbound HA trimers (Figs 1 and 2). These findings suggest that loss of conformational flexibility of the A Helix plays an important role in the mechanism of CR6261-mediated neutralization of HA. In agreement, Fab CR6261 has been shown to neutralize HA by impeding the pH-dependent conformational rearrangements of the A Helix and the fusion peptide that are required for fusion^22^. Moreover, a previous HDX-MS study determined that structural dynamics of the fusion peptide and neighboring regions increases as it approaches activation in response to decreased pH, and fusion peptide release was proposed to be the initial step for HA activation^39^. Thus, the decreased conformational flexibility of the conserved fusion machinery we report here is likely the molecular mechanism underlying impediment of activation-related conformational rearrangements that lead to antibody mediated neutralization of HA. In summary, HDX-MS analysis of HA-Fab CR6261 complexes not only identifies the main antigenic site, but also reveals the changes in conformational dynamics associated with neutralization of HA trimers in solution.

### HDX-MS analysis of diverse drug candidates targeting different HA subtypes reveals a molecular indicator of broad neutralization

**Figure 3:**
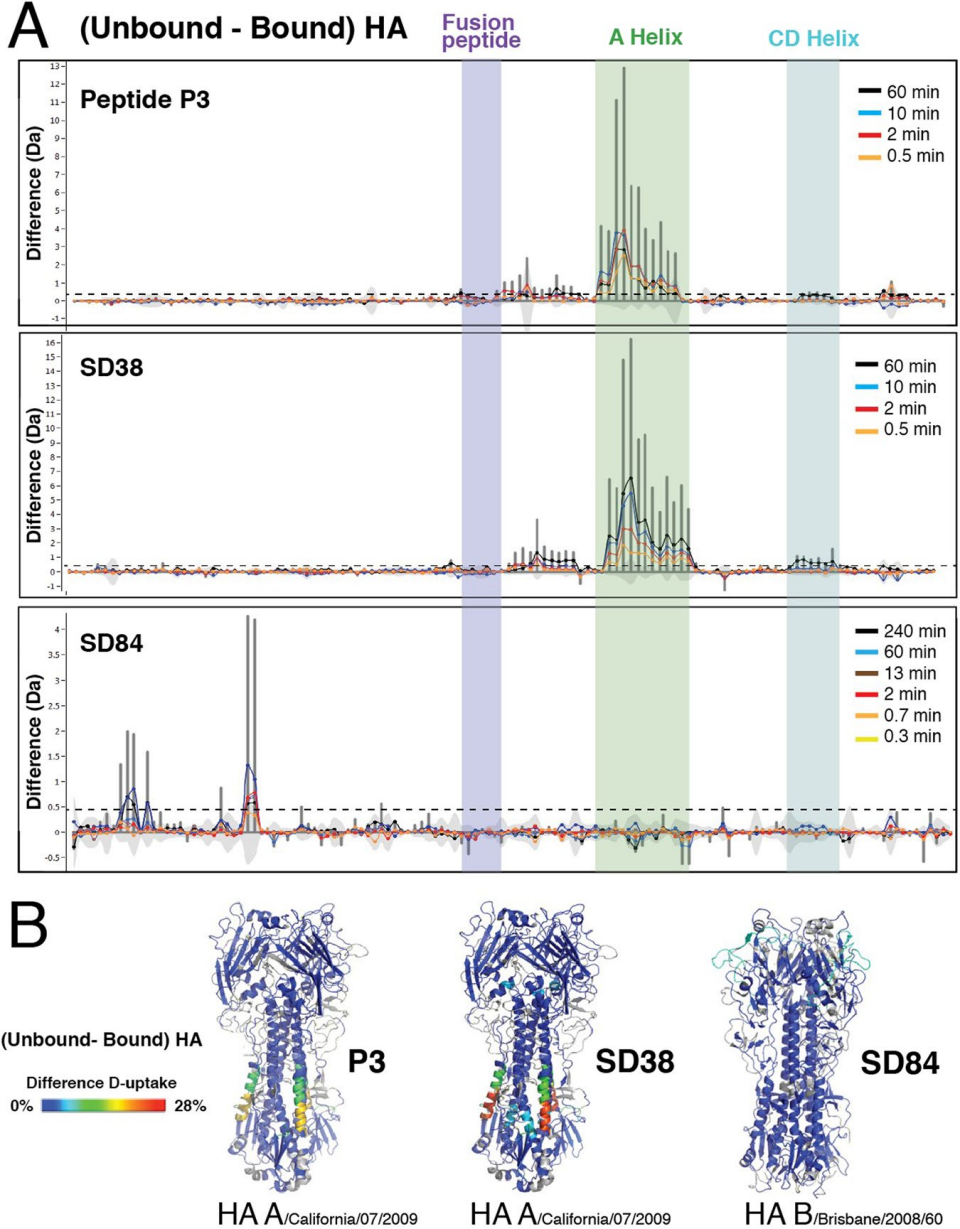
HDX-MS analysis of diverse drug candidates in complex with different HA subtypes. HDX-MS analysis of the broadly neutralizing HA stem binding molecules P3 and SD38 was performed on HA A California/07/2009 strain, whereas HA B/Brisbane/2008/60 strain was used for SD84, a head binding drug candidate that only recognizes HA-B strains. (**A**) Bar plots representing the difference in deuterium uptake for peptides originating from HA in the presence or absence of each drug molecule reveal the distinct effects of these drug candidates on HA. Each bar along the X-axis represents a unique HA peptide from the peptide library (ordered from N- to C- terminus based on the first amino acid of each peptide), and the Y-axis represents the total difference in D uptake for a given peptide. Light grey shades show the differential uptake errors for each peptide, and a black horizontal line represents the threshold above which deuterium uptake differences between free and bound HA were considered significant. This threshold corresponds to three times the average standard deviation between triplicate repeats, and is approximately 0.3 Da for all measured peptides. (**B**) The deuterium uptake difference between bound and unbound HA is projected on the trimeric structure of HA A/California/07/2009 (PDB: 3UBQ) for cyclic peptide P3 and SD38, and HA/B/Brisbane/2008/60 (PDB: 4FQM) for SD84. The differences in deuteration are represented in a rainbow color scheme, where blue corresponds to HA regions that are not affected by binding of the drug candidate, while red corresponds to 28% difference. HA regions that are not covered in our peptide library are shown in grey.

**Figure 4:**
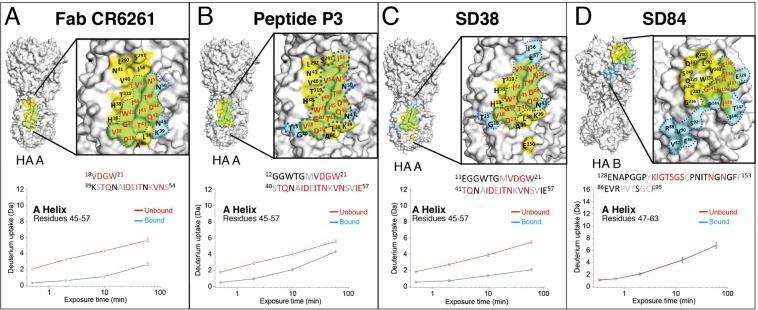
HDX-MS accurately determines the epitope for each drug molecule and reveals distinct effects on the conformational dynamics of HA. The epitopes identified by HDX-MS for (**A**) Fab CR6261, (**B**) Cyclic Peptide P3, (**C**) Llama single domain SD38 and (**D**) Llama single domain SD84 are mapped onto the molecular surface representation of the corresponding HA strain. For each drug candidate, the areas identified as protected by HDX-MS were plotted in blue, and the residues shown to mediate contacts by X-Ray crystallography were mapped in yellow. Thus, the green area is the overlap between the epitopes identified by X Ray co-crystallography and HDX-MS, revealing that these methods generate similar results in all cases. The close-up view shows all amino acids identified as part of the epitope by X-Ray crystallography and HDX-MS with the residues identified by both methods highlighted in red. The PDB identifiers for the X-Ray co-crystal structures of the different drug candidates in complex with different HA strains are 3GBN (Fab-CR6261-HA A/Brevig Mission/1/1919), 5W6I (P3-HA A/Puerto Rico/8/1934), 6FYT (SD38 –HA A/Salamon Islands/3/2006) and 6CNV (SD84 –HA B/Brisbane/2008/60), respectively. The precise amino-acid sequences identified as the epitope by HDX-MS are also shown with residues identified by X-Ray crystallography colored in red, while those buried within the protein structure and therefore not accessible from the protein surface are shown in grey. Below, the deuterium uptake graphs of peptides originating from the A Helix are shown for the corresponding drug candidate. Peptides stemming from the A Helix in HA A/California//07/2009 strain (HA2 residues 45–57, Panels A–C) and the HA B/Brisbane/2008/60 strain (HA2 residues 47–63, Panel D) are essentially indistinguishable, demonstrating conservation of the unique conformational dynamics of the A Helix across distantly related HA subtypes. The deuterium incorporation graphs upon binding of each drug candidate show that the 3 broadly neutralizing HA stem binding drug candidates (Fab CR6261, P3 and SD38) significantly inhibit conformational breathing of the A Helix, whereas the strain specific HA head-binder SD84 does not. Furthermore, the deuterium incorporation rates of each drug candidate are distinct and reveal significantly larger protection upon binding of Fab CR6261 and SD38 than P3.

We employed our semi-automated HDX-MS method optimized for HA to characterize the epitope of HA in complex with two broadly neutralizing drug molecules known to bind a similar epitope on the HA stem: the cyclic peptide P3 and the llama single domain antibody SD38. As expected, the global difference plots of our HDX-MS results show that, like Fab CR6261, both molecules induce protection of the region directly adjacent to the fusion peptide, and the A Helix (Fig. 3). Mapping of these differences onto the atomic model of HA immediately reveals the interaction area, and analysis of the overlapping peptides in these regions identifies residues 12–21 and 40–57 as the epitope for P3, and residues 11–21 and 41–57 for SD38 (Figs 3 and 4). Thus, the epitope determined by HDX-MS comprises 28 residues, of which 15 and 13 are found to make contacts in the co-crystal structures of P3 (PDB: 5W6I) and SD38 (PDB: 6FYT), respectively. Similarly to Fab-CR6261, 7 additional contact residues identified by X-ray crystallography belong to regions around the glycosylation sites of HA1, which are not covered by peptic peptides, and are therefore not detected by HDX-MS (Fig. 4). In both cases, however, this semi-automated HDX-MS epitope mapping method optimized for HA robustly determined the main recognition sites within a day, highlighting its value for drug discovery pipelines of anti-HA molecules.

Importantly, HDX-MS is currently the only technology available for studying the local conformational dynamics of antigenic complexes of HA in solution, as these complexes are too large for NMR studies. Interestingly, our HDX-MS data reveal that the 3 broadly neutralizing drug candidates studied here prevent conformational breathing of the A Helix to different extents: After 60 min of labeling SD38 decreases deuterium uptake by 28%, while Fab CR6261 and P3 decrease uptake by 20 and 15%, respectively (Figs. 2B and 3B). Analysis of the peptides in this region reveals distinct uptake profiles and shows that SD38 almost completely abolishes deuterium incorporation at all time points, whereas deuterium uptake in the presence of P3 is considerably higher, especially at later time points (Fig. 4). The consistency of our data for unbound HA across different experiments, along with the reproducibility between replicates in HA bound to the different drug candidates, further validate the significance of the distinct deuterium uptake profiles observed in the A Helix (Fig. 4). Intriguingly, a third area (residues 93–117 from HA2) showed small, but statistically significant protection at longer labeling times upon binding of SD38, and, to a lesser extent, P3 (Figs. 3 and 4). Intrinsic deuterium incorporation for this region is minimal, because this region corresponds to the central part of the highly ordered CD helix, which is buried in the inter-subunit interface of the HA trimer (Figs 1 and S2). Thus, the consistent differences observed in the CD Helix reflect changes in deuterium incorporation that cannot be attributed to direct contacts with SD38, but rather to an indirect allosteric effect of binding (Fig. 3). Taken together, our HDX-MS data reveal that different drug candidates targeting a similar epitope in the HA stem induce distinct direct and allosteric changes upon binding. These differences can be due to a number of factors, such as different binding kinetics and affinities, and our findings may guide downstream pharmacological development.

Highly variable viral antigens like HA present an additional challenge for drug discovery pipelines, because the analytical tools developed for one strain may not be transferable to distantly related homologs. For instance, we optimized our HDX-MS method for a type A HA strain, but sequence identity between type A and B subtypes is extremely low (30%). To test the application breadth of our HDX-MS method for HA, we probed the interaction between an HA-B strain and SD84, a single domain llama antibody that only recognizes HA-B strains. Despite very low sequence conservation, we obtained excellent coverage of the HA/B/Brisbane/2008/60 strain except for the areas around the glycosylation sites of HA1. Thus, the HDX-MS workflow did not require any optimization and was directly applicable for epitope mapping studies on HA-B subtype strains (Fig. 3). Interestingly, we found that the conformational flexibility we identified for the A Helix in the HA-A strain is almost identical in the HA-B strain, revealing conservation of this unique trait across distantly related HA subtypes (Fig. 4). These findings further suggest that the highly dynamic behavior of the A Helix reported by HDX-MS is important for its functional role in mediating fusion. HDX-MS also shows that, unlike the broadly neutralizing anti-HA molecules, SD84 has no effects on the HA stem region and does not alter conformational dynamics of the A and CD helices (Figs 3 and 4). Instead, binding of this subtype specific molecule induces protection of two amino acid regions in the highly glycosylated HA head (HA1 residues 129–153 and 86–95) (Fig. 3). Notably, deuterium uptake differences between free and SD84-bound HA were significantly lower relative to the changes detected in the A helix upon binding of the different stem binders (Fig. 3). We reasoned that this was likely due to the low deuterium uptake of these regions in comparison to the highly dynamic A Helix. To validate the significance of these relatively smaller changes we included additional time points in our HDX-MS data set and found that these differences were highly consistent for all time points and repeats (Fig. 3). Comparison with X-ray crystallographic data confirmed that the two regions identified by HDX-MS were indeed part of the epitope of SD84 (Fig. 4D). Although some contacts could not be identified due to suboptimal peptide coverage in the glycan-rich areas of the HA head, no “false-positives” were found and our results validate the feasibility of HDX-MS to report interactions in the proximity of HA glycan moieties (Fig. 4D). These results demonstrate that our HDX-MS method for HA enables analysis of the conformational impact of different drug molecules on distantly related HA subtypes, even in areas with low intrinsic deuterium incorporation.

In summary, we optimized a semi-automated HDX-MS method for HA that is widely applicable to study highly divergent HA subtypes and their interactions with diverse drug candidates, including large protein therapeutics and small synthetic peptides. In fact, once an efficient digestion protocol was established, HDX-MS functioned as a robust, higher throughput epitope-mapping alternative, determining the main recognition site of each drug candidate within a day. Moreover, our HDX-MS data for free and drug-bound HA offer unique insights into the local conformational dynamics of these large antigenic complexes with important implications for drug discovery. Our data for unbound HA reveal an unexpectedly high conformational flexibility of the A helix, the main antigenic target for broadly neutralizing anti-HA molecules. We further show conservation of the highly dynamic behavior of the A Helix across distantly related HA subtypes, which suggests that flexibility of the A Helix is required for the pH-dependent conformational switching that enables HA function. Moreover, all broadly neutralizing drug candidates tested here significantly inhibited conformational breathing of the A Helix, whereas the HA head binder did not (Fig. 4). Thus, HDX-MS reveals a major change in the local conformational dynamics of the A Helix that has critical mechanistic implications and is associated with broad neutralization. As a result, these specific changes are an important indicator of broad neutralization that can serve as a marker for broad anti-HA activity of new drug molecules. Together, our findings highlight the potential for HDX-MS analysis to identify promising broadly neutralizing drug candidates against HA at early stages of drug discovery^40,41^.

## Supplementary information

Supplementary Figures

## References

[1] Becker W, Bhattiprolu KC, Gubensak N, Zangger K (2018). Investigating Protein-Ligand Interactions by Solution Nuclear Magnetic Resonance Spectroscopy. Chemphyschem: a European journal of chemical physics and physical chemistry.

[2] Monaco S, Tailford LE, Juge N, Angulo J (2017). Differential Epitope Mapping by STD NMR Spectroscopy To Reveal the Nature of Protein-Ligand Contacts. Angewandte Chemie.

[3] Davies DR, Cohen GH (1996). Interactions of protein antigens with antibodies. Proc Natl Acad Sci USA.

[4] Kong L (2015). Complete epitopes for vaccine design derived from a crystal structure of the broadly neutralizing antibodies PGT128 and 8ANC195 in complex with an HIV-1 Env trimer. Acta crystallographica. Section D, Biological crystallography.

[5] Long F (2015). Cryo-EM structures elucidate neutralizing mechanisms of anti-chikungunya human monoclonal antibodies with therapeutic activity. Proc Natl Acad Sci USA.

[6] Ward AB, Wilson IA (2017). The HIV-1 envelope glycoprotein structure: nailing down a moving target. Immunol Rev.

[7] Sevy AM (2013). Epitope mapping of inhibitory antibodies targeting the C2 domain of coagulation factor VIII by hydrogen-deuterium exchange mass spectrometry. J Thromb Haemost.

[8] Berkowitz SA, Engen JR, Mazzeo JR, Jones GB (2012). Analytical tools for characterizing biopharmaceuticals and the implications for biosimilars. Nature reviews. Drug discovery.

[9] Deng B, Lento C, Wilson DJ (2016). Hydrogen deuterium exchange mass spectrometry in biopharmaceutical discovery and development - A review. Analytica chimica acta.

[10] Iacob RE, Engen JR (2012). Hydrogen exchange mass spectrometry: are we out of the quicksand?. J Am Soc Mass Spectrom.

[11] Masson GR, Jenkins ML, Burke JE (2017). An overview of hydrogen deuterium exchange mass spectrometry (HDX-MS) in drug discovery. Expert opinion on drug discovery.

[12] Engen JR, Wales TE (2015). Analytical Aspects of Hydrogen Exchange Mass Spectrometry. Annual review of analytical chemistry.

[13] Tu T (2010). Protein-peptide affinity determination using an h/d exchange dilution strategy: application to antigen-antibody interactions. J Am Soc Mass Spectrom.

[14] Kochert BA, Iacob RE, Wales TE, Makriyannis A, Engen JR (2018). Hydrogen-Deuterium Exchange Mass Spectrometry to Study Protein Complexes. Methods in molecular biology.

[15] Wei H (2014). Hydrogen/deuterium exchange mass spectrometry for probing higher order structure of protein therapeutics: methodology and applications. Drug discovery today.

[16] Marciano DP, Dharmarajan V, Griffin PR (2014). HDX-MS guided drug discovery: small molecules and biopharmaceuticals. Current opinion in structural biology.

[17] Claesen J, Burzykowski T (2017). Computational methods and challenges in hydrogen/deuterium exchange mass spectrometry. Mass spectrometry reviews.

[18] DynamX HDX Data Analysis Software 3.0 (2018).

[19] Sautto GA, Kirchenbaum GA, Ross TM (2018). Towards a universal influenza vaccine: different approaches for one goal. Virology journal.

[20] Corti D (2011). A neutralizing antibody selected from plasma cells that binds to group 1 and group 2 influenza A hemagglutinins. Science.

[21] Dreyfus C (2012). Highly conserved protective epitopes on influenza B viruses. Science.

[22] Ekiert DC (2009). Antibody recognition of a highly conserved influenza virus epitope. Science.

[23] Ekiert DC (2011). A highly conserved neutralizing epitope on group 2 influenza A viruses. Science.

[24] Kashyap AK (2008). Combinatorial antibody libraries from survivors of the Turkish H5N1 avian influenza outbreak reveal virus neutralization strategies. Proc Natl Acad Sci USA.

[25] Nakamura G (2013). An *in vivo* human-plasmablast enrichment technique allows rapid identification of therapeutic influenza A antibodies. Cell host & microbe.

[26] Sui J (2009). Structural and functional bases for broad-spectrum neutralization of avian and human influenza A viruses. Nat Struct Mol Biol.

[27] Throsby M (2008). Heterosubtypic neutralizing monoclonal antibodies cross-protective against H5N1 and H1N1 recovered from human IgM + memory B cells. PLoS One.

[28] Brandenburg B (2013). Mechanisms of hemagglutinin targeted influenza virus neutralization. PLoS One.

[29] Sparrow E, Friede M, Sheikh M, Torvaldsen S, Newall AT (2016). Passive immunization for influenza through antibody therapies, a review of the pipeline, challenges and potential applications. Vaccine.

[30] Laursen, N. S. *et al*. Universal Protection against Influenza Infection by a Multi-Domain Antibody to Influenza Hemagglutinin. *In press* (2018). 10.1126/science.aaq0620 PMC6241527 30385580

[31] Fleishman SJ (2011). Computational design of proteins targeting the conserved stem region of influenza hemagglutinin. Science.

[32] Koday MT (2016). A Computationally Designed Hemagglutinin Stem-Binding Protein Provides *In Vivo* Protection from Influenza Independent of a Host Immune Response. PLoS pathogens.

[33] Whitehead TA (2012). Optimization of affinity, specificity and function of designed influenza inhibitors using deep sequencing. Nature biotechnology.

[34] Kadam RU (2017). Potent peptidic fusion inhibitors of influenza virus. Science.

[35] Memczak H (2016). Anti-Hemagglutinin Antibody Derived Lead Peptides for Inhibitors of Influenza Virus Binding. PLoS One.

[36] Laursen NS (2018). Universal protection against influenza infection by a multidomain antibody to influenza hemagglutinin. Science.

[37] Trabjerg E, Kartberg F, Christensen S, Rand KD (2017). Conformational characterization of nerve growth factor-beta reveals that its regulatory pro-part domain stabilizes three loop regions in its mature part. The Journal of biological chemistry.

[38] Zhang Z, Smith DL (1996). Thermal-induced unfolding domains in aldolase identified by amide hydrogen exchange and mass spectrometry. *Protein science: a publication of the Protein*. Society.

[39] Garcia NK, Guttman M, Ebner JL, Lee KK (2015). Dynamic changes during acid-induced activation of influenza hemagglutinin. Structure.

[40] Zhang HM (2010). Simultaneous reduction and digestion of proteins with disulfide bonds for hydrogen/deuterium exchange monitored by mass spectrometry. Anal Chem.

[41] Pradzinska M (2016). Application of amide hydrogen/deuterium exchange mass spectrometry for epitope mapping in human cystatin C. Amino acids.

