# Supplementary material for: Epitope mapping of diverse influenza Hemagglutinin drug candidates using HDX-MS
Source: Sci Rep. 2019 Mar 18;9:4735. doi: 10.1038/s41598-019-41179-0 (PMC6427009; doi:10.1038/s41598-019-41179-0)
Supplement: Supplementary file 1 — Supplementary Figures [file 41598_2019_41179_MOESM1_ESM.docx]

# Supplementary Figures

# Epitope mapping of diverse influenza Hemagglutinin drug candidates using HDX-MS

Cristina Puchades, Başak Kűkrer, Otto Diefenbach, Eveline Sneekes-Vriese, Jarek Juraszek, Wouter Koudstaal and Adrian Apetri^*^

# Janssen Vaccines and Prevention, Janssen Pharmaceutical Companies of Johnson & Johnson, Archimedesweg 6, 2333 CN, Leiden, the Netherlands.

*Correspondence to: [AApetri@its.jnj.com](mailto:AApetri@its.jnj.com)

**FIGURE S1**

**
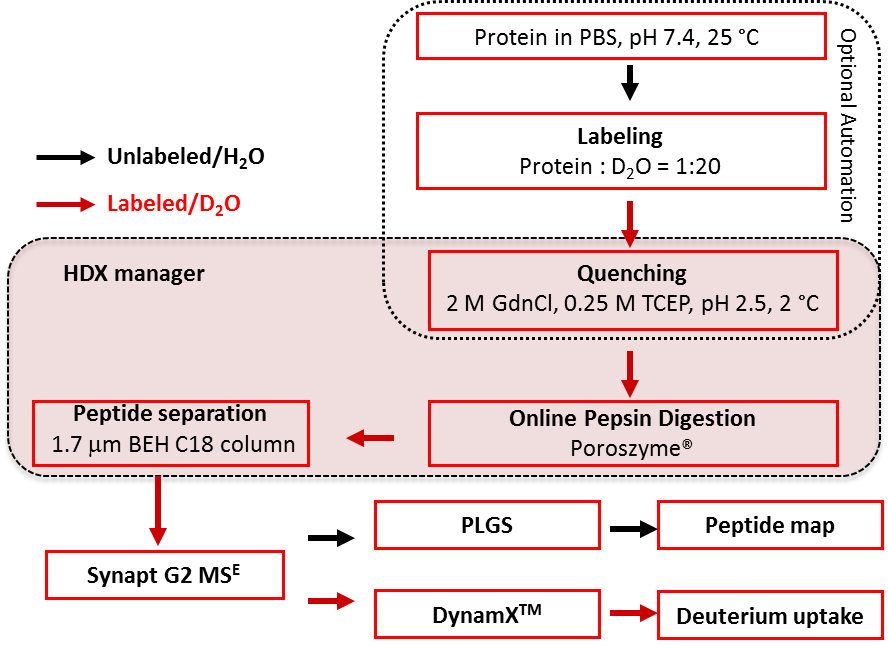
**

**Figure S1. Semi-automated HDX-MS experimental workflow**. Free HA and drug-bound HA stocks in PBS are diluted 20-fold in PBS buffered D_2_O and incubated for 0.5, 2, 10 or 60 minutes at 25 ^0^C. Labeling is quenched by lowering the temperature to 2 ^0^C at low pH in the presence of high concentrations of denaturants and reducing agents and proteins are subjected to online pepsin digestion. Due to the low pH and temperature during the quench step, TCEP is typically the reducing agent of choice, and very high concentrations (up to 0.5 M TCEP) are often used in HDX-MS experiments [^40^](#_ENREF_40)^,^[^41^](#_ENREF_41). The peptic peptides are separated and analyzed by LC-MS and data processed using PLGS and DynamX^TM^ software. Non-deuterated controls are used for peptide identification.

**FIGURE S2**


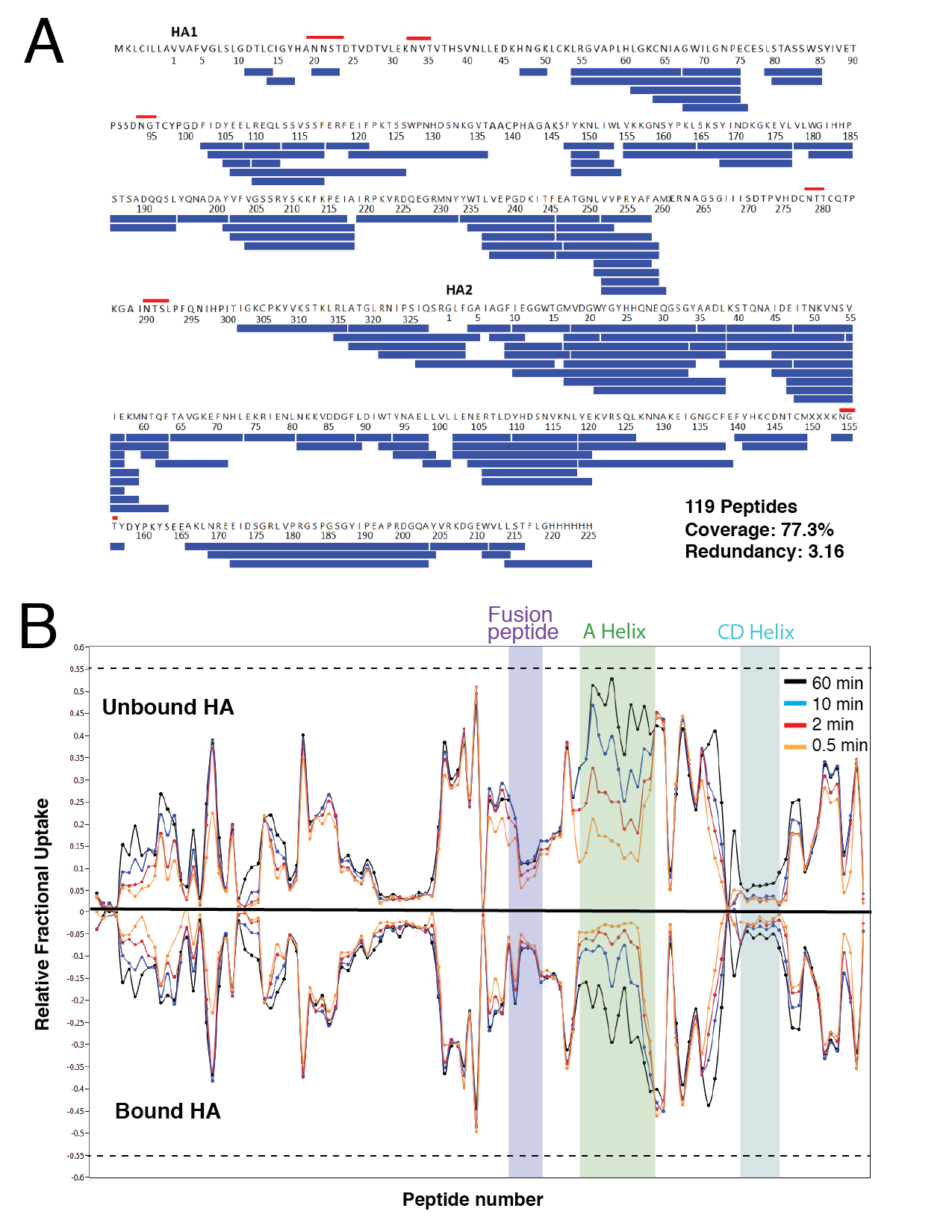


**Figure S2.** **Quality of the HDX-MS data for HA.** **A.** Blue bars represent the peptic peptides of HA A/California/07/2009 reproducibly detected after HDX labeling experiments. Red lines indicate the theoretical N-glycosylation sites on the HA molecule, which remain largely uncovered. **B.** Butterfly plot showing the relative fractional deuterium uptake per peptide for each labeling time point for free and Fab CR6261 bound HA. Overlapping peptides stemming from the same regions present very similar deuterium uptake profiles validating the HDX-MS data. For instance, all peptides originating from the buried and highly structured CD Helix remain almost undeuterated throughout the course of the experiment, whereas peptides from the A Helix present a distinctive deuterium incorporation profile, and have the highest levels of deuterium uptake of the entire HA molecule at t=60min. Changes in deuterium incorporation upon Fab binding result in a loss of symmetry in the butterfly plot, and localize to the A Helix and the region directly adjacent to the fusion peptide. Based on the measured level of deuterium uptake in internal peptide controls under our experimental conditions we estimate an average ~45% back-exchange (highlighted by dashed black lines).

**FIGURE S3**


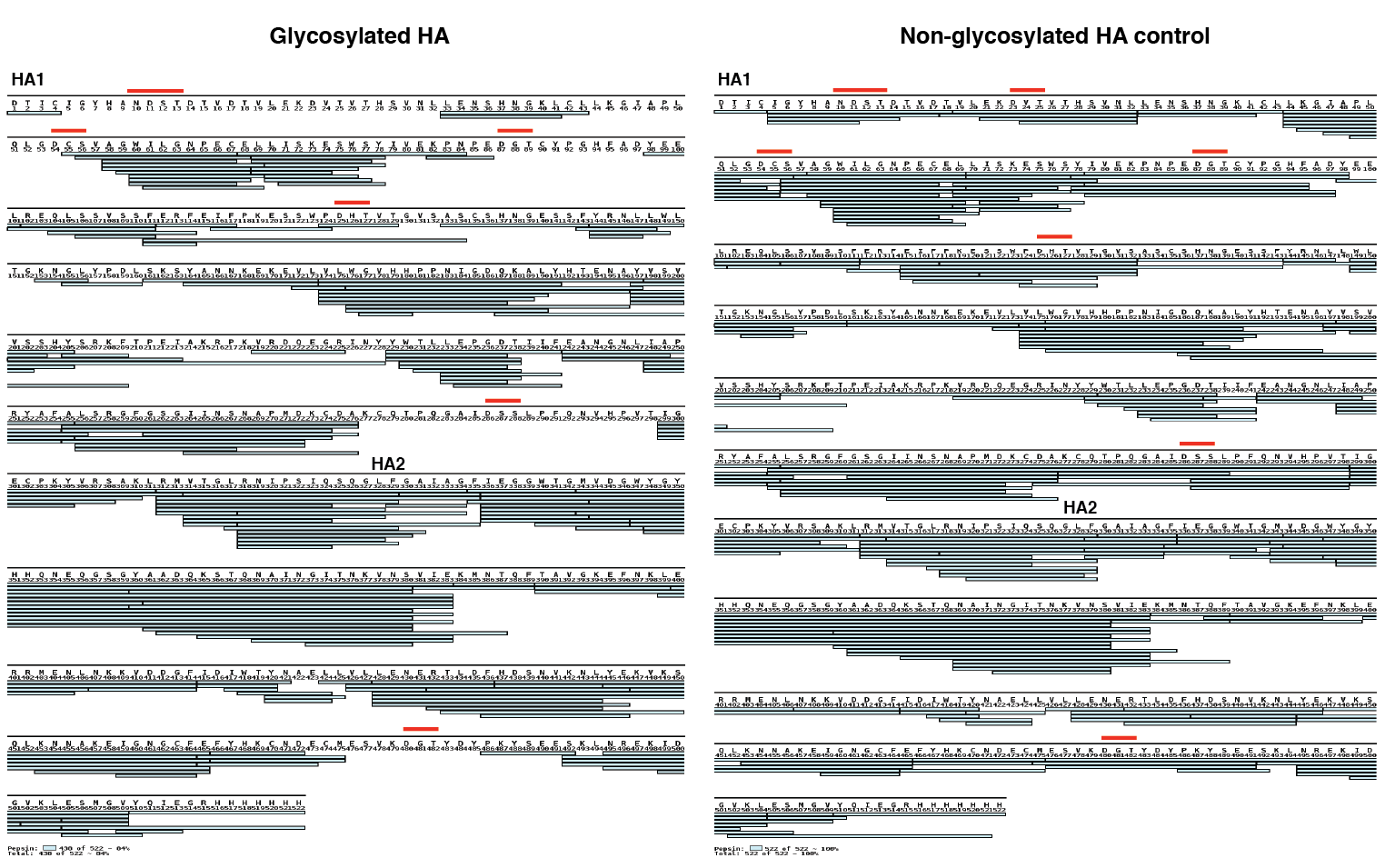


**Figure S3.** **The effect of HA glycosylation on sequence coverage**. Peptide coverage map, where each peptic peptide reproducibly detected in undeuterated HA is represented by a bar under the corresponding amino acid sequence. Red lines indicate the theoretical N-glycosylation sites on the HA molecule, and these regions remain largely uncovered in glycosylated HA (left) but are fully covered after removal of the glycan moieties via PNGase treatment (right).

**FIGURE S4**

**Figure S4. Stoichiometry of Fab CR6261 binding to trimeric HA**. SEC-MALS chromatogram of Fab CR6261 (blue), HA A/California/07/2009 (green) and a 1.1: 1 mixture (expressed in mols of monomer units) of Fab CR6261 and HA (red) confirms complex formation under HDX-MS experimental conditions. The molecular weight of the complex shows that virtually all HA molecules are bound to three Fab CR6261 molecules per HA trimer.
